# Case Report: Autologous Bone Marrow Derived Intrathecal Stem Cell Transplant for Autistic Children - A Report of Four Cases and Literature Review

**DOI:** 10.3389/fped.2021.620188

**Published:** 2021-10-06

**Authors:** Georg S. Kobinia, John J. Zaknun, Christof Pabinger, Brenda Laky

**Affiliations:** ^1^Austrian Society of Regenerative Medicine, Vienna, Austria; ^2^Institute of Regenerative Medicine (IRM), Graz, Austria; ^3^Primary Pediatric Care Centre – Kinderkompetenzzentrum, Vienna, Austria

**Keywords:** autologous, stem cell transplant (SCT), bone marrow (BM), intrathecal, point-of-care method, safety, autism, case report

## Abstract

Despite steadily growing numbers of children diagnosed with autism spectrum disorders (ASD), causative treatment is unavailable. Recently, biological cell therapies involving pluripotent cells have raised hopes towards sustained beneficial outcome. We herein report data of four children diagnosed with ASD, who were treated with autologous, bone marrow (BM)-derived, intrathecally and simultaneously intravenously applied, point-of-care stem cell transplant (SCT). The three boys and one girl received the diagnosis at ages between 2–4 years. The decision to perform the procedure was preceded by limited beneficiary impact of conventional symptom-based, psychological and pharmacological interventions. At ages of 4–14 years the children received their SCT, no immediate or late adverse events were reported. Disappearance of symptoms were observed by the parents during the following year and consequently improved Autism Treatment Evaluation Checklist (ATEC) scores were reported. The SCT procedure, in trained hands, can be a safe and promising treatment option in children with ASD, responding in a non-satisfactory manner to conventional treatments. It is postulated that SCT may, among others, assert its positive effect by counteracting a cerebral inflammatory autoimmune process which in turn supports the responsiveness to behavioral and pharmacological interventions. Our results in this small group are encouraging, but certainly need further investigation in larger cohorts.

## Introduction

Treating and raising children with ASD poses an enormous burden. Despite the fact that infantile autism has been first described by Leo Kanner (1) as early as 1943, yet the etiology of ASD is not completely understood. The genetic background of ASD comprises heterogenic traits (2) that are described to be responsible for affecting the brain network signal conduction. However, causative treatment options are still not available. The disease shows an increase in incidence with climbing numbers up to one out of 54 children (3). While progress has been made in establishing a psychological and symptom adapted treatment, the search for biologic diagnostic parameters of the disease has become a major topic in recent ASD research.

Several groups from Sweden, UK, and the USA provided valuable insight to understand the genetic background and risk factors for ASD (4–8). While some groups postulate that ASD are highly familial (7, 8), a final verdict on the underlying cause of ASD is still far away (2).

A milestone in ASD research was the finding that ASD may be induced by an interplay between genetic predisposition and immunological inflammatory factors (9–11). This changed paradigm of ASD conception opened the door to introduce biological (autologous or allogenic) stem cell therapies known among others for their immune-modulating properties (12, 13). In this context, an increasing number of published investigations reporting on treatment attempts employing either autologous or allogeneic bone marrow (BM) or umbilical derived pluripotent cells (14–19) have encouraged us to report the results in a pilot group of children. Autologous BM-derived, point-of-care stem cell transplantation (SCT) was used due to the fact that it carries virtually no risk with respect to adverse autoimmune reaction and for its universal availability, as it does not exclude children without stored umbilical cord or access to umbilical cord blood.

Two studies applying intrathecal instillation of autologous stem cells were reported by Sharma et al. (14) from India and Thanh et al. (15) from Vietnam in small cohorts. To start a new procedure in a geographical and political part of the world as large as the EU in our opinion warrants the report of small case studies, which undoubtedly should and will be followed by larger studies in due time. Thus our aim was to contribute to the limited experience acquired so far by adding two factors to the described operative protocol: namely i.v. application of stem cells and the transfusion of bone marrow derived plasma, known to be rich in cytokines, and growth factors.

## Methods

All children received state-of-the-art, non-invasive treatment as suggested by their specialists before and after undergoing autologous, point-of-care SCT. For this retrospective case report, outcome was reported by the parents including the Autism Treatment Evaluation Checklist (ATEC) score, which was completed online (https://www.autism.org/autism-treatment-evaluation-checklist/). The ATEC, a questionnaire developed by the Autism Research Institute (San Diego, CA) (6), is widely used in publications to describe changes over time. ATEC total scores range from 0 to 179 points and are determined by adding up the four sections (section I (0–28 points): speech/language/communication; section II (0–40 points): sociability; section III (0–36 points): sensory/cognitive awareness; and section IV (0–75 points): health/physical/behavior). A higher score indicates a higher level of impairment.

The objective evaluation regarding progress and improvements in ASD encounters numerous difficulties. While there are already various diagnostic tests (e.g., Autism Diagnostic Observation Schedule, ADOS; Childhood Autism Rating Scale, CARS; Autism Diagnostic Interview - Revised, ADI-R), progress and improvement are even more difficult to assess. Diagnostic tests, such as ADOS, generally require specialists for execution and the test per se was not developed to test for changes over time. Consequently, we decided to rely on the ATEC score with all its limitations and advantages as has been done in other publications in this field (16).

All SCTs were performed as point-of-care procedures in a class IIa operating room with sterile air flow. The following standard operating procedure (SOP) was used: (1) anesthesia was prepared with rectal administration of Midazolam (1mg/kg body weight with max. of 15mg); (2) slowly starting sedation with 5–8ml (i.v.) 1%-Propofol (sedoanalgesia); (3) positioning of the patient on one side following surgical washing and draping the anterior and posterior iliac crest; (4) injection of 2ml of 1%-Xylocaine at the planned puncture sites on the periosteum and subcutaneously; (5) aspiration of BM from the posterior and anterior iliac crest followed by a transfer of the BM aspirate to a sterile blood bag; (6) the BM aspirate was then processed in the operating room according to the proprietary protocol using a fully automated cell separator (Sepax S-100; Biosafe S.A., Eysins, CH); (7) after lumbar puncture of the dural sac 2ml of cerebral spinal fluid (CSF) was routinely withdrawn in order to prevent high intrathecal pressure secondary to injection of the stem cell concentrate; (8) intrathecal administration of the obtained BM concentrate (~1ml/10kg body weight); and (9) i.v. administration of the remaining BM concentrate and plasma supernatant (10). Standard postoperative care was applied.

Samples of BM aspirate/CSF and concentrate were transferred to the same laboratory immediately after SCT and were analyzed with fluorescence activated cell sorter (FACS) using a stem cell kit from Beckman Coulter and the ISHAGE protocol (https://www.bc-cytometry.com/PDF/DataSheet/IM3630.pdf). Stem cell counts including CD34+ and CD45+/leukocytes, which are known as indirect indicators for progenitor cells, were obtained to quantify stem cells.

A summary of demographic, diagnostic, and laboratory data and stem cell transplantation details of the four children with ASD are presented in Table 1.

**Table 1:** Demographic, diagnostic, and laboratory data and stem cell transplantation details of the four children with autism spectrum disorder (ASD).

	**Case #1**	**Case #2**	**Case #3**	**Case #4**
Gender	Male	Male	Male	Female
Age at time of diagnosis of autism	4 y	3 y	4 y	2 y
Diagnostic details	F84.0 (ADOS = 13) + F90.0 + F79 neuropsychiatrist	F84.0 (CARS = 28.5) + F82 neuropsychiatrist	F84.0 (CARS = 38.5) + F90.0 + F80.0 pediatric psychiatrist	F84.1 (CARS = 25) + F80.1 + F71 pediatric neurologist
Therapy before and/or after autologous SCT	speech therapy, medication	ABA, speech therapy, medication, nut. suppl.	medication, nut. suppl., horse therapy	ABA
Age and body weight at time of first procedure	7 y2 m, 29 kg	7 y5 m, 32 kg	14 y4 m, 74 kg	3 y8 m, 17.5 kg
**Bone marrow related details**				
BM aspirate (ml/kg body weight)	1.55	1.40	0.81	1.42
CD-34 cell concentration in BM aspirate (μl^−1^)	302	406	182	330
CD-34 cell concentration in BM concentrate (μl^−1^)	600	1,040	950	1,090
CD-45 mononuclear cells in BM aspirate (nl^−1^)	11.9	31.2	12.9	23.0
CD-45 mononuclear cells in BM concentrate (nl^−1^)	22.4	144.0	62.1	59.4
SC concentrate (ml/kg body weight)	0.28	0.31	0.14	0.57
**Stem cells related details**				
Intrathecal vol. of SC concentrate instillation	3.0 ml	4.0 ml	5.0 ml	2.5 ml
Intrathecal number of CD-34 cells injected	1.8 x 10^6^	4.16 x 10^6^	4.75 x 10^6^	2.73 x 10^6^
Intravenous vol. of SC-concentrate injected	5.0 ml	6.0 ml	5.0 ml	7.5 ml
Intravenous number of CD-34 cells injected	3 x 10^6^	6.24 x 10^6^	4.75 x 10^6^	8.18 x 10^6^
‡SC viability in BM aspirate (%)	92.9	89.1	66.0	92.6
‡SC viability in SC concentrate (%)	87.0	90.0	57.3	90.2

*ABA, Applied Behavior Analysis Therapy; ADOS, Autism Diagnostic Observation Schedule; ASD, autism spectrum disorder; BM, bone marrow; CARS, Childhood Autism Rating Scale; F71, moderate mental retardation; F79, unspecified mental retardation; F80.0, specific speech articulation disorder; F80.1, expressive language disorder; F82, specific developmental disorder of motor function; F84.0, childhood autism; F84.1, atypical autism; F90.0, Disturbance of activity and attention; m, months; nut. suppl., nutritional supplements; OP, operation/surgery; SC, stem cell; SCT, stem cell transplantation; y, years.*

‡ *viability as determined 24–36h after harvesting.*

## Case Description

### Case #1

The birth, a planned caesarean section in the 38^th^ week of pregnancy, of a boy from a 40-year-old healthy mother and a 41-year-old healthy father, went without complications. The birth body weight and length were 3340g and 51cm, respectively. The child developed normally, like his older brother, until the age of 2 years. Thereafter, the parents noticed that the boy gradually lost the vocabulary he had already acquired without comorbidity. At 2.5 years of age the child was non-verbal; thus, speech therapy was started. Since the speech therapy showed no success after about 1.5 years of implementation, the boy was evaluated in a special clinic for psychiatry and diagnose with autism by Autism Diagnostic Observation Schedule (ADOS) ASD at the age of 4-years.

Immediately after the diagnosis, the child began the so-called ABA (Applied Behavior Analysis) therapy at home (3h/day). Drug treatment with Atomoxetine (8mg oral/day), Risperidone (1mg/ml from 0.5 to 2 x 0.5ml/day), and Cerebrolysin® injection 2 x 2.5ml/week was not started until 6 months after diagnosis. Despite moderate cognitive improvements, there was no change in speech according to the mother and the child was weaned from all medication. Finally, the parents decided to let the child undergo autologous SCT in our center.

**Figure 1:**
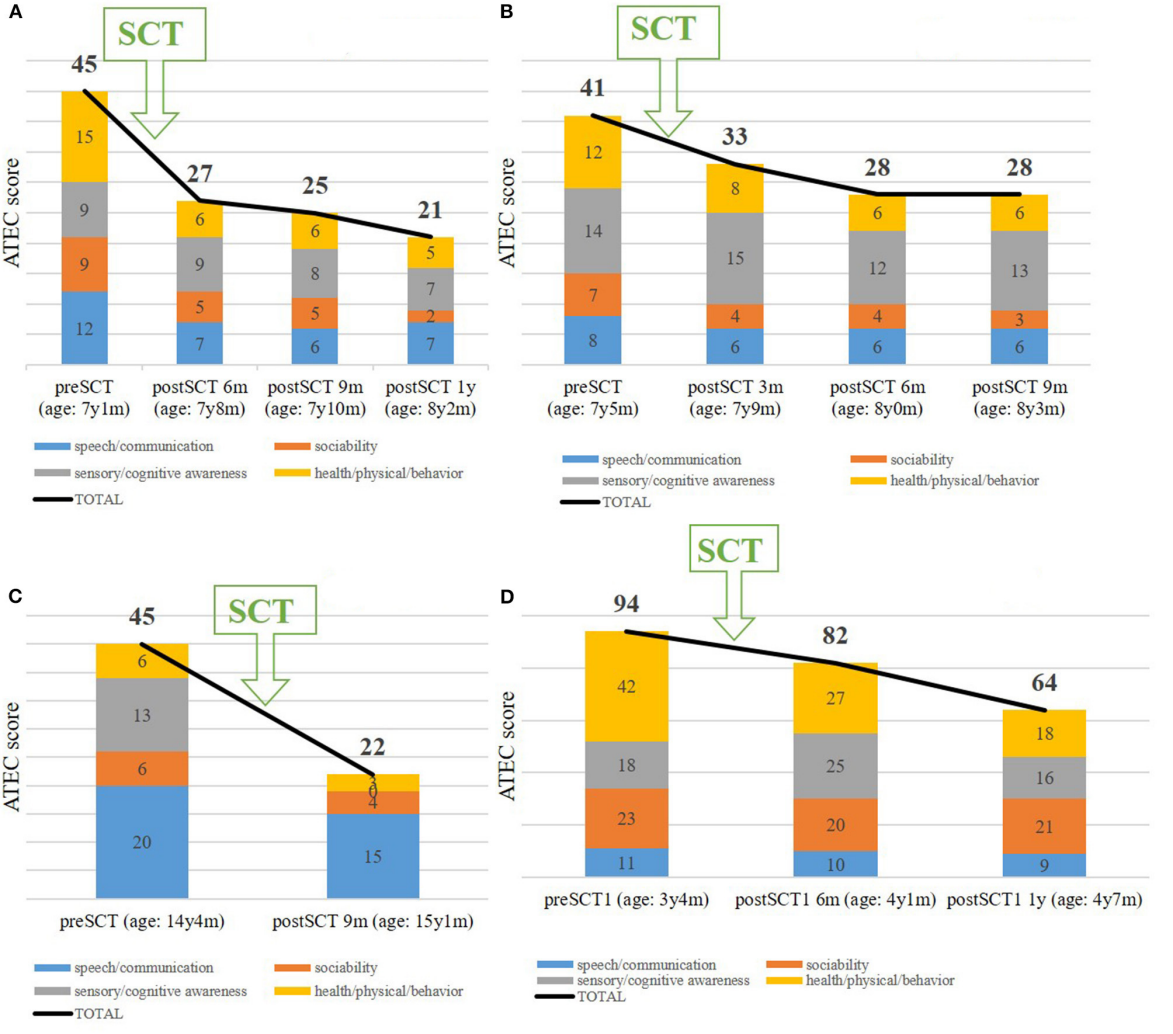
Autism treatment evaluation checklist (ATEC) scores of **(A)** case #1, **(B)** case #2, **(C)** case #3, and **(D)** case #4 before and after stem cell Children's stem cell transplantation (SCT).

The boy showed no minor or serious side effects. The parents observed first impressions of a benefit of the SCT at the 3 months follow-up assessment. After a year the child improved mainly regarding speech (e.g., the child began to formulate simple sentences with several words, to ask meaningful questions, and finally to have conversations), social behavior (e.g., he showed better eye contact, obeyed better, and had no more tantrums), and also behavior regarding food intake and hyperactivity. All available, parents-generated ATEC scores are presented in Figure 1A.

### Case #2

The single child of a healthy 25-year-old mother and a healthy 31-year-old father was born in the 39^th^ week of pregnancy via caesarean section due to umbilical cord malposition around the neck of the child. The birth weight was 3600g and the body length 51cm. The first signs of an ASD (e.g., did not respond to name, shows little eye contact) were already apparent in the first year of life. At the age of three the boy was then diagnosed as autistic (F84.0) by a neuropsychiatrist. Dyspraxia (F82) was also found. Drug treatment with Cerebrolysin® injection 1-5ml/week, as well as behavior therapy (2h/day) was applied as recommended by the specialist for a few years. At the age of 7.5, the language development was retarded to the level of a 3 year old child. Based on recommendations BM-derived SCT of and exchanges with other parents, the boy's parents decided to have their son treated with autologous SCT.

The child had no minor or serious side effects. Parents observed significant behavioral changes regarding hyperactivity, rigid behavior, but also improvements with original digestive problems, sensitivity to noise, and anxiety 9 months after the autologous SCT. Significant behavioral changes regarding hyperactivity, rigid behavior, but also improvements with original digestive problems, sensitivity to noise, and anxiety were reported by the parents 9 months after the autologous SCT. All available, parents-generated ATEC scores are presented in Figure 1B.

### Case #3

The boy was born as a single child to a 30-year-old healthy mother and a 35-year-old healthy father *via* caesarean section due to umbilical cord malpositioning around the neck. The birth body weight was 4,000g. The mother noticed that her baby boy showed hardly any eye contact, did not react to names, and showed hardly any social interaction. However, a definitive diagnosis of ASD was made by a psychiatrist later on at the age of 3 years. The child lagged proper speech development; according to the parents, he understands everything, but cannot express himself. He was not treated with any drugs nor behavioral therapy as advised by his physicians. The decision of the parents to go for autologous SCT was made at the boy's age of 14.5 years and was also based on personal communication with other parents.

The boy showed no minor or serious side effects. Despite the advanced age, linguistic (e.g., he began to speak two-word sentences) and especially sensory improvements such as perceiving and reacting to the environment were noticed by the parents. All available, parents-generated ATEC scores are presented in Figure 1C.

### Case #4

The only girl in this case series was born as the little sister of a 2-year older boy to a 34-year-old healthy mother and a 31-year-old healthy father via an elective caesarean section at the 38^th^ week of pregnancy. The birth weight was 3,180g and the body length 50cm. The girl developed normally, like his older brother, until the age of 1.5 years, thereafter the parents noticed that her behavior was not the same as the older sibling (e.g., she was not reacting when called, no eye contact, not communicating, and she always wanted to be left alone). With 18 months the child was diagnosed as autistic by a neurologic pediatrician. Additionally, the girl was diagnosed with bronchial asthma requiring inhalation therapy. Immediately after diagnosis, the child started ABA, which included 3h/day with a specialized psychotherapist and the remaining time ABA was continued with her mother. Because of only slight improvements with 2-years of ABA therapy, the parents decided to let the child undergo autologous SCT. Deficits existed primarily regarding speaking, frustration, and concentration.

SCT was performed without any minor or major complications. 3 months after the SCT, the girl showed improved initiative and learning behavior and was even able to remember the learned matter. Furthermore, she has no asthma anymore and the numbers of colds she used to have, significantly decreased. All available, parents-generated ATEC scores are presented in Figure 1D.

Furthermore, parents reported that they were very much satisfied (Case #1 and Case #3) or much satisfied (Case #2 and Case #4) with the procedure and that they would undergo SCT again. This satisfaction correlates with the ATEC score. Considering an ATEC total score between 31 and 50 points as a moderate form of ASD and values below 30 points as a mild form, then ASD all cases changed from moderate to mild form of ASD possibly in relation to autologous SCT (Figures 1A–D). No relapse has been observed so far (1–2 years).

## Discussion

With this report we present promising results regarding safety and efficacy suggesting application of autologous, BM-derived, intrathecally and simultaneously intravenously applied, point-of-care SCT in four children with ASD.

### Immediate and Delayed Complications

Using sedation and local anesthesia, the procedure involving BM-biopsy and intrathecal instillation of isolated and concentrated BM stem cells resulted in no immediate or delayed minor or major adverse events. This is most likely attributed to stringent condition present in the operation theatre and expertise of the surgeon and anesthetist. The post-operative course during the following 48h was uneventful too as preventive non-steroidal anti-inflammatory drug pre-medication and anti-emetics were prescribed.

Sharma et al. (14) in a group of 34 children reported asides minor side effects including nausea, vomiting, and local pain, a percentage of major adverse events in 31% (10/32) comprising hyperactivity and epileptic seizures. Thanh et al. (15) reported no major adverse event in similarly sized study on 30 children undergoing repeated interventions. In our pilot study we encountered neither immediate, nor delayed major adverse events.

### Assessment of Treatment Outcome

In our small series, treatment outcome reported over a follow-up period of 1–1.5 year relied on the feedback of the parents and ATEC score. We are aware that parents reporting outcome is subject to bias and to a certain extent on behavioral non-compliance of the children, in our context and similar to a randomized controlled trial using ATEC as primary endpoint (16), parental-reported ATEC proved to be reliable to assess longitudinal outcome.

Despite the presence of professional rating tools such as ADOS, CARS, and ADI-R to diagnose and evaluate ASD, such tools may have drawback in daily practice. In addition, the dependency on various sources to acquire a more complete clinical picture has been advocated (17).

Diagnostic tests are basically not well suited to evaluate changes over time and as such do require specialists for execution. Especially in patients with ASD and daily fluctuations, parental assessment with their continuous observation of behavioral and developmental changes, may be a major advantage to single time point assessments.

However, one confounder is that neither standardized test nor parental observations can account for the inherent nature of ADS symptoms and their improvements over time. In our series, the enrolment of the children to undergo the SCT procedure was motivated by the frustration of parents a previous lack of improvement.

### Efficacy of the Procedure

Following SCT, reported ATEC scores revealed significant improvements in all ATEC subgroups including speech/communication, social behavior, sensory/cognitive awareness, health/physical/behavior compared to pretreatment status. Such amelioration not only improved the immediate quality of life of the children and their environment, but may also contribute to the children's future ability to conduct an independent life in a protected environment. Hence, from an economical point of view, such improvements may lead to significant reductions of continuously incurring care costs with age.

Comparable to our treatment approach applying autologous BM-derived intrathecally applied SCT, two investigations -one applying a single (14) and the other two- intrathecal (15) stem cell instillations also reported encouraging results. Mixed results were reported using intravenous delivery of either, autologous or allogenic, umbilical cord blood-derived SCT. Two studies reported significant improvements (18, 19), while the remaining two only a trend towards improvement in a sub-analysis in children without intellectual disability (20, 21). Details hereto are summarized in Table 2. Intrathecal application was favored by us on these clinical and also theoretical grounds. Stem cells are too large to cross the blood brain barrier, hence, they must be applied directly into the CSF *via* intrathecal route in order to reach the brain.

**Table 2:** Published investigations reporting intrathecal or intravenous application of either autologous or allogenic stem cell transplantation for children with autism spectrum disorder (ASD).

**Before 2020**	**Sharma et al. (14)**	**Lv et al. (18)**	**Dawson et al. (19)**	**Chez et al. (20)**
Journal (year)	Stem Cells 2013	J of Transl Med 2013	Stem Cells Transl Med 2017	Stem Cells Transl Med 2018
Study design	Open label proof-of-concept, 24 centers	Controlled, non-randomized, (Phase I/II)	Single-center phase I, open-label	Placebo-controlled crossover
Patients (n) (gender, age)	32 (8f/24m; 3-33y)	37 (1f/36m; 3-12y)	25 (4f/21m; 2-6y)	29 (4f/25m; 2-7y)
Groups	32 intervention	14 UCB 9 UCB+UC 14 controls (therapy)	25 intervention	14 intervention 15 placebo
SC origin	BM	UCB and UC	UCB	UCB
Application route	Intrathecal	Intravenous (4x)	Intravenous (1x)	Intravenous (1x)
Follow-up	26 months	4, 8, 16, 24 weeks	6 and 12 months	12 and 24 weeks
Safety	Minor AE 17.9% vomiting 10.7% nausea 7.1% pain (injection) 7.1% pain (aspiration) 3.6% spinal headache Major AE 6 transient increase In hyperactivity 3 seizures 1 persistent 6 months Increase in hyperactivity	No major AE 3 minor AE (low grade fever)	9 related AE 5 allergic reaction 2 agitation 1 aggression 1 other psychiatric disorder No serious AE	3 “probable” AE 2 renal/urinary disorders 1 constitutional symptom 14 “possible” AE 8 gastrointestinal disorders 4 renal/urinary disorders 2 constitutional symptom No serious AE
Efficacy	Sign. improvements of scores (ISAA, CGI, FIM, Wee-FIM)	Both intervention groups showed sign. improvements (CGI, CARS, ABC) compared to controls	Sign. improvements of scores (CGI, EOWPVT-4, PDDBI, EGT)	Trend towards improvement of scores (EOWPVT-4, ROWPVT-4, SBFR/SBKN, ABC, CGI)
**After 2020**	**Thanh et al. (15)**	**Dawson et al. (21)**	**Sharifzadeh et al. (22)**	**Sharma et al. (23)**
Journal (year)	Stem Cells Trasl Med 2020	J Pediatr 2020	Asia Pac Psychiatry 2021	Am J Stem Cells 2020
Study design	Open label BMT repeated within 6 months	2:1 randomized, placebo-controlled, double-blind (Phase II)	Randomized controlled trial	Open label non-randomized
Patients (n) (gender, age)	30 (5f/25m; 3-7.4y)	180 (37f/143m; 2-7y)	32 (5f/27m; 5-15y)	254 (31f/223m; 2-34y)
Groups	30 intervention	56 autologous UCB 63 allogeneic UCB 61 placebo	14 intervention (plus rehabilitation) 18 control (rehabilitation therapy and risperidone)	254 intervention (plus neurorehabilitation)
SC origin	BM	UCB	BM	BM
Application route	Intrathecal (2x)	Intravenous	Intrathecal (2x)	Intrathecal
Follow-up	6, 12, and 18 months	6 and 12 months	6 and 12 months	Mean 7.5 months
Safety	No major AE	84 minor AE 29 placebo 55 UCB 16 infusion reactions 4 placebo 12 UCB 6 serious /moderate AE 3 placebo 3 UCB	No serious AE No other AEs	No major AE Efficacy
Efficacy	CARS: 50 to 46.5 VABS: 53.6 to 60.5 Improved social communication, language, and daily skills	Trend towards improvement in the allogenic UCB group (CGI)	Limited clinical efficacy (no differences regarding CARS total score, GARS-II autism index, and CGI global improvement)	Positive change in ISAA and CARS; improved brain metabolism with PET-CT scan in all 86 patients

*ABC, Aberrant Behavior Checklist; AE, adverse event; BM, bone marrow; CARS, Childhood Autism Rating Scale; CGI, Clinical Global Impression scale; EGT, Eye Gaze Tracking of Social Stimuli; EOWPVT-4, Expressive One-Word Picture Vocabulary Test (4^th^ edition); FIM, Functional Independence Measure; f, female; GARS-II, Gillian Autism Rating Scale (2^nd^ edition); ISAA, Indian Scale for Assessment of Autism; m, male; PET-CT, Positron emission tomography; PDDBI, Pervasive Developmental Disorder Behavior Inventory; ROWPVT-4, Receptive One Word Picture Vocabulary Test (4^th^ edition); SBFR, Stanford Binet (5^th^ edition) Fluid Reasoning; SBKN, Stanford Binet (5^th^ edition) Knowledge subtests; sign, significant; SCT, stem cell transplantation; UC, umbilical cord; UCB, umbilical cord blood; VABS-3, Vineland Adaptive Behavior Scales (3^rd^ edition)*.

An important detail reported while interviewing the parents was that children started to respond much better and quicker to their speech and behavior therapy 3–6 months following SCT. Published data suggest that SCT reduces the immunological inflammatory disease of the brain associated with ASD and thus, opens the door for effectiveness of ABA and speech therapy. Indeed, a recent proteomic analysis study discovered nine serum proteins to be significantly different in ASD compared to typically developing boys and a significant correlation with ASD severity according to ADOS (24). Possible mechanisms for the way stem cells improve autism have been discussed more extensively by Liu et al. (25). In summary, two mechanisms seem to prevail: (a) reset of the immunological system and (b) improved vascular perfusion of the brain, two mechanisms that are addressed both by the stem cells but also by the bone marrow plasma, rich in growth hormones.

There is a large body of data pointing at immune-related genetically coupled risk factors and events associated with ASD. A cascade of events, leading disruption of neuronal maturation and dysfunctional networking through dysfunctional astrocytic neuronal support. A comprehensive review on this topic was recently published by Liu et al. (25). More interestingly, neuropathological investigations have recently provided evidence in support of the inflammatory theory, describing perivascular lymphocytic infiltration in the cerebral white, gray matter, and neuronal leptomeninges, this infiltrates were quantitatively accompanied by a corresponding magnitude of astrocytic activation in the affected regions of the brain. In Addition they reported significant loss on neurons and glial cells of the cerebral gray matter immediately adjacent to the leptomeningeal space. Brain micropathology also involved periventricular and other cerebral spinal fluid brain interfaces and vascular ependymal structures, all contributing to a functional disruption of the blood brain barrier (26, 27).

However, in ASD it seems that the overexpression of specific histocompatibility (HLA) genes (chromosome 6) and particularly activating KIR genes (chromosome 19) play an important role in promoting the cellular autoimmune cascade in brain tissue (28). The overexpression of the genes as compared to the general population provides a molecular basis for understanding events triggering a pathological immune response to viral or microbial antigens. BM-derived SCT is capable of targeting these pathological processes in the brain without having immediate and mid-term adverse events. The longevity of the effect of BM-derived SCT on suppressing inflammation and derailed autoimmune processes in the central nervous system (29) requires further investigations in larger cohorts. Furthermore, it is speculated if repeated treatment may have a cumulative effect on ASD. In addition, long-term observation are needed in children following autologous BM-derived SCT, though low likelihood, to rule out potential undesirable complications. Addition issues that require further elucidation involve (a) the efficacy and safety of employed cell types i.e., allogenic vs. autologous, umbilical cord-derived vs. autologous BM-derived, (b) the route of administration (intravenous vs. intrathecal), and (c) the added value of injecting BM-derived plasma. Presently, the rather limited available literature indicates more favorable results when employing intrathecal over intravenous route, probably because with the later, most of the cells will be filtered by the lung parenchyma during their first blood passage.

Autologous SCT have a biological advantages over allogenic stem cells and resemble a novel and promising treatment option for autistic children and adolescent not benefiting from conventional symptom-based and behavioral therapy. In ASD affected children providing intrathecal SCT at an earlier age should be associated with a higher benefit, as the brain plasticity and neurogenesis are at their maximum (30, 31), while perivascular damage to the neuronal circuitry is minimal.

## Conclusions

Previous findings indicate that autologous, BM-derived, intrathecally and simultaneously intravenously applied, point-of-care SCT is a safe therapeutic option by showing no adverse events. Furthermore, our findings also showed improvements in all four ATEC subsets including speech/communication, social behavior, sensory/cognitive awareness and health/physical behavior. Our and previous results by other authors are promising, but mandate further investigations in a larger controlled cohort of patients including objective methods such as biomarkers to possibly better understand the underlying individual dysfunction and potentially allow a stratification of those patients who may benefit most from this treatment strategy.

## Data Availability Statement

The original contributions presented in the study are included in the article/supplementary material, further inquiries can be directed to the corresponding author/s.

## Ethics Statement

Ethical review and approval was not required for the study on human participants in accordance with the local legislation and institutional requirements. Written informed consent to participate in this study was provided by the participants' legal guardian/next of kin. Written informed consent was obtained from the individual(s), and minor(s)' legal guardian/next of kin, for the publication of any potentially identifiable images or data included in this article.

## Author Contributions

GK and BL: wrote the first draft of the manuscript and coordinated and supervised data collection. GK: an experienced general and cardiac surgeon, performed all SCTs. GK, JZ, CP, and BL: substantially contributed to interpretation of data for the work and critically reviewed and revised the manuscript for important intellectual content. All authors gave their final approval and agreed to be accountable for all aspects of this work ensuring its integrity and accuracy.

## Conflict of Interest

The authors declare that the research was conducted in the absence of any commercial or financial relationships that could be construed as a potential conflict of interest.

## Publisher's Note

All claims expressed in this article are solely those of the authors and do not necessarily represent those of their affiliated organizations, or those of the publisher, the editors and the reviewers. Any product that may be evaluated in this article, or claim that may be made by its manufacturer, is not guaranteed or endorsed by the publisher.
